# The R-S difference index: A new electrocardiographic method for differentiating idiopathic premature ventricular contractions originating from the left and right ventricular outflow tracts presenting a left bundle branch block pattern

**DOI:** 10.3389/fphys.2022.1002926

**Published:** 2022-09-19

**Authors:** Lei Zhao, Ruibin Li, Jidong Zhang, Ruiqin Xie, Jingchao Lu, Jinming Liu, Chenglong Miao, Wei Cui

**Affiliations:** ^1^ Department of Cardiology, The Second Hospital of Hebei Medical University, Shijiazhuang, China; ^2^ The Second Hospital of Hebei Medical University, Shijiazhuang, China

**Keywords:** electrocardiogram, premature ventricular contractions, septal right ventricular outflow tract, diagnostic index, aortic sinus cusp

## Abstract

**Introduction:** Differentiating idiopathic premature ventricular contractions (PVCs) originating from the right and left ventricular outflow tracts with a left bundle branch block (LBBB) morphology is relevant to catheter ablation planning and important for lowering the risk of complications. This study established a novel electrocardiographic (ECG) criterion to discriminate PVCs originating from the septum of the right ventricular outflow tract (s-RVOT) and those originating from the aortic sinus cusp of the left ventricular outflow tract (LVOT-ASC).

**Methods:** A total of 259 patients with idiopathic PVCs originating from ventricular outflow tract with a LBBB pattern who underwent successful catheter ablation were retrospectively included. Among them, the PVCs originated from the s-RVOT in 183 patients and from the LVOT-ASC in 76 patients. The surface ECGs of the PVCs and sinus beats were analyzed using an electronic caliper. The R-S difference index in the precordial leads was calculated as V2R + V3R + V4R − V1S.

**Results:** PVCs originating from both the s-RVOT and LVOT-ASC displayed an inferior axis (dominant R waves in leads II, III, and aVF). Compared with the s-RVOT group, the R-wave amplitudes on leads II, III, and aVF were significantly larger in the LVOT-ASC group (*p* < 0.001, *p* < 0.003, and *p* < 0.001, respectively). Compared to the LVOT-ASC group, the s-RVOT group showed smaller R-wave amplitudes on leads V1–V6 (*p* = 0.021, *p* < 0.001, *p* < 0.001, *p* < 0.001, *p* < 0.001, and *p* < 0.001, respectively) and larger S-wave amplitudes on leads V1–V3 (*p* < 0.001, *p* < 0.001, and *p* < 0.001, respectively). Lead V3 was the most common transitional lead in both groups. Analysis of the receiver operating characteristic curve showed that the R-wave amplitude on lead V3 had the largest area under the curve (AUC) of 0.856 followed by the R-wave amplitudes on leads V4 (0.834) and V2 (0.806). The AUC of the R-S difference index was 0.867. An R-S difference index greater than 20.9 predicted an LVOT-ASC origin with 73.7% sensitivity and 86.3% specificity. This index is superior to previous criteria in differentiating PVCs with LBBB morphology and inferior axis originating from s-RVOT vs. LVOT-ASC.

**Conclusions:** The R-S difference index in precordial leads is a useful new ECG criterion for distinguishing LVOT-PVCs from RVOT-PVCs with LBBB morphology.

## Preface

Idiopathic ventricular arrhythmias are a common clinical arrhythmogenic condition that includes PVCs and ventricular tachycardia (VT). PVCs of ectopic origin can originate from multiple sites of the right and left ventricles. Previous study shows that the outflow tracts are the most common origin of idiopathic PVCs, and 60%–80% of idiopathic PVCs originate from the RVOT. (Nikoo et al., 2020) The 12-lead electrocardiogram of PVCs with different origins have distinctive features. However, PVCs that are anatomically adjacent have similar body-surface ECG patterns. The RVOT and LVOT are anatomically adjacent, with the RVOT spiraling around the left anterior aspect of the LVOT. Thus, the pulmonary valve is located in the left anterior part of the aortic valve and is more horizontally positioned than the aortic valve (Enriquez et al., 2019). The aortic sinus is located in the center of the heart and consists of three leaflets: the left coronary cusp (LCC), the right coronary cusp (RCC), and the non-coronary cusp (NCC). The most common site of PVCs originating from the aortic sinus is the LCC followed by the RCC and the junction between the LCC and RCC (Yamada et al., 2013). The base of the aortic sinus is a continuation of the fibrous tissue between the aortic valve and the mitral valve. NCC is a rare site of ventricular arrhythmias because it does not come into direct contact with the ventricular myocardium (Ioannidis et al., 2021). Catheter ablation is a micro incision procedure and is an effective treatment for recurrent ventricular arrhythmias with a success rate of 80%–100%, making it the treatment of choice for symptomatic ventricular arrhythmias (Lerman, 2015). With the widespread use of catheter ablation in clinical practice, the preoperative prediction of PVC origin based on body-surface ECG has become clinically important, especially for discriminating left and right ventricular PVCs. Clinically, during the radiofrequency catheter ablation of PVCs, the femoral vein approach is generally chosen for RVOT PVCs, whereas the femoral artery approach is used for LVOT PVCs. Therefore, the accurate preoperative localization of PVC origin based on ECG can help optimize the surgical procedure, reduce puncture injuries, and reduce the risk of surgical and puncture-related complications.

The outflow tract (OT) is located in the highest part of the ventricle, thus, OT-PVCs show a large and tall R-wave in leads II, III, and aVF. This feature, in combination with the characteristic V1 patterns and the precordial transitional lead (the first precordial leads with R/S>1), is used to preliminarily distinguish PVCs between LVOT and RVOT. In general, PVCs originating from RVOT have an ECG pattern of the LBBB. In contrast, PVCs originating from the LVOT usually show a right bundle branch block (RBBB) pattern (Yamada, 2019). Notably, because the aortic sinus is anatomically adjacent to the septum of the RVOT, some PVCs originating from the aortic sinus also exhibit a LBBB pattern in lead V1. As a result, the ECG shows (i) a large and tall R-wave pattern in leads II, III, and aVF and (ii) an LBBB pattern, making it difficult to discriminate PVCs of LVOT and RVOT. In this study, we statistically analyzed the electrocardiographic indices of PVCs of the left and right ventricular outflow tracts and developed a method to discriminate PVCs originating from s-RVOT and the LVOT-ASC based on the electrocardiographic parameters.

## Materials and methods

### Study subjects

A total of 259 patients with idiopathic PVCs originating from the outflow tract with a LBBB pattern who underwent successful radiofrequency ablation in the catheterization unit of the Second Hospital of Hebei Medical University from January 2015 to January 2019 were included in this study. Among which, 183 patients (54 males and 129 females) with PVCs originating from s-RVOT and 76 patients (41 males and 35 females) with PVCs originating from LVOT-ASC. All patients were admitted to the hospital for ECG, chest X-ray, and echocardiography. No significant abnormalities were found on completion of relevant laboratory tests. All patients underwent 24-h ambulatory electrocardiograms and reported frequent PVCs (more than 10,000 beats in 24 h). The study followed the principles of the Declaration of Helsinki and was approved by the Ethics Committee of the Second Hospital of Hebei Medical University (ethics number: 2022-P025).

### Electrophysiological study

The PVC origin was confirmed by intra-cardiac electrophysiological study in all patients. All patients underwent electrophysiological study after five half-lives of antiarrhythmic drug withdrawal. If PVCs were absent, isoproterenol (1.0–3.0 μg/min) was administered intravenously until constant and measurable PVCs were obtained. The right femoral vein was routinely punctured, and a cold saline perfusion ablation catheter (ThermoCool ablation catheter, Biosense Webster, United States) or ST large-tip catheter was delivered *via* an 8F vascular sheath to the RVOT; for the LVOT, the catheter was delivered retrograde via femoral artery puncture. The target site for RFCA was determined by a combination of activation mapping (earliest local activation time preceding QRS onset by ≥20 ms) during PVCs/IVTs and pace mapping (pacing marker was identical to the QRS pattern in ≥ 11 leads of the 12-lead ECG of a clinical PVC) (Yamada et al., 2007).

### Radiofrequency ablation

After the ideal target was marked, the perfusion ablation catheter was applied to discharge the ablation at a power of 30–35 W. The temperature was set at 43°C, and the discharge test was carried out for 10–20 s. The disappearance of PVCs after discharge was the marker of effective ablation. The disappearance of the PVCs was followed by 90–120 s of continuous ablation. Acute success of ablation was indicated by the following three criteria: (i) after the disappearance of the PVCs due to ablation, isoprenaline was administered intravenously, and no further isomorphic PVCs were observed within 30 min; (ii) no clinical PVCs were indicated upon the postoperative review of the ECG; and (iii) no clinical PVCs were indicated upon postoperative bedside ECG monitoring for 24 h.

### Electrocardiographic analysis

ECGs are recorded with a paper speed of 25 mm per second. The morphology of QRS and Q-, R- and S-wave amplitudes of sinus beat and PVCs in each lead of the standard 12-lead ECG were measured and statistically analyzed.

### Statistical treatment

All data were statistically analyzed using SPSS 25.0 software (SPSS Inc., Chicago, IL, United States). Measurement data with normal and chi-squared distributions were described as mean ± standard deviation, and groups were compared via independent sample t-test. Other measurement data were described as median (quartiles), and non-parametric rank-sum test was used for comparison between groups. Count data were expressed as frequency and percentage, and the X^2^ test was used for between-group comparisons. A joint model was developed using logistic regression analysis. The receiver operating characteristic (ROC) curve and the AUC were used to compare the accuracy of the predictive value of each scoring model. *p* < 0.05 was considered to indicate statistically significant differences.

## Results

### Clinical characteristics about the research subjects

Based on the site of PVC origin, the enrolled patients included 183 cases in the s-RVOT group (54 males and 129 females, mean age = 46.13 ± 14.42 years) and 76 cases in the LVOT-ASC group (41 males and 35 females, mean age = 48.54±14.29 years). The s-RVOT group included more women than men (female-to-male ratio = 2.39), while the LVOT-ASC group included more men than women (female-to-male ratio = 0.85). The gender difference between the two groups was statistically significant (*p* < 0.001). There was no obvious difference in age between the two groups (*p* = 0.220). In the s-RVOT group, the red blood cell count, hemoglobin, creatinine, left ventricular end-diastolic diameter, interventricular septal thickness, and left ventricular posterior wall thickness were lower than in the LVOT-ASC group (*p* = 0.011, 0.022, 0.014, 0.012, 0.008, and 0.004, respectively). Clinical characteristics including age, gender, laboratory results, and echocardiographic parameters of the two groups is shown in Table 1.

**TABLE 1:** Comparison of the baseline characteristics betweenw the s-RVOT and LVOT-ASC groups.

Variables	s-RVOT (*n* = 183)	LVOT-ASC (*n* = 76)	*p*-value
Age (years)	46.13±14.42	48.54±14.29	0.220
Gender			
Male	54 (29.5)	41 (53.9)	<0.001
Female	129 (70.5)	35 (46.1)	
Red blood cell count	4.47 (4.15,4.78)	4.68 (4.33,4.91)	0.011
White blood cell count	6.2 (5.3,7.43)	6.4 (5.4,7.2)	0.838
Hemoglobin	136 (125,143)	140 (128,149.75)	0.022
Total cholesterol	4.18 (3.61,4.87)	4.28 (3.72,4.7)	0.88
Creatinine	61.2 (53.68,70.23)	66.75 (55,74.53)	0.014
Serum potassium	4.04 (3.84,4.26)	3.99 (3.74,4.28)	0.281
Left ventricular ejection fraction (EF%)	62.41 (61.47,63.73)	61.88 (60.87,63.64)	0.225
Left atrial diameter (mm)	33 (30,35)	34 (30.25,37)	0.100
Left ventricular end diastolic diameter (mm)	47 (45,49.75)	48 (46,52)	0.012
Interventricular septal thickness (mm)	9 (8,10)	10 (9,10)	0.008
Left ventricular posterior wall thickness (mm)	9 (8,10)	10 (9,10)	0.004

### Electrocardiographic characteristics

The morphology of QRS of PVCs in both groups exhibited tall, upright R waves in leads II, III, and aVF. However, the R-wave amplitude in leads II, III, and aVF were significantly greater in the LVOT-ASC group than the s-RVOT group (*p* < 0.001, *p* < 0.003, and < 0.001, respectively). The R-wave amplitudes in leads V1–V6 in the s-RVOT group were significantly smaller than those in the LVOT-ASC group (*p* = 0.021, *p* < 0.001, *p* < 0.001, *p* < 0.001, *p* < 0.001, *p* < 0.001, *p* < 0.001, respectively). The S-wave amplitudes in leads V1–V3 were significantly greater than those in the LVOT-ASC group (*p* < 0.001). There were no significant differences in the duration of the QRS wave between the two groups of PVCs (Table 2).

**TABLE 2:** Comparison of the electrocardiographic measurements of the s-RVOT and LVOT-ASC groups.

	s-RVOT (*n* = 183)	LVOT-ASC (*n* = 76)	*p*-value
Lead I			
Q amplitude PVC (mV)	0 (0,0)	0 (0,0)	0.376
R amplitude PVC (mV)	1.6 (0.8,3.4)	2.5 (1.1,4.45)	0.017
S amplitude PVC (mV)	0 (0,1)	0 (0,1.38)	0.282
QRS duration PVC (ms)	112 (100,120)	112 (97,120)	0.744
Lead II			
Q amplitude PVC (mV)	0 (0,0)	0 (0,0)	0.121
R amplitude PVC (mV)	17 (14,19)	20.05 (15.85,23)	<0.001
S amplitude PVC (mV)	0 (0,0)	0 (0,0)	0.516
QRS duration PVC (ms)	120 (116,120)	120 (112,123)	0.656
Lead III			
Q amplitude PVC (mV)	0 (0,0)	0 (0,0)	0.045
R amplitude PVC (mV)	15.9 (13,18.5)	17.45 (14.6,22.5)	0.003
S amplitude PVC (mV)	0 (0,0)	0 (0,0)	0.964
QRS duration PVC (ms)	120 (112,120)	120 (112,124)	0.375
Lead aVR			
Q amplitude PVC (mV)	9.1 (7.5,10.8)	11 (9.18,13)	<0.001
R amplitude PVC (mV)	0 (0,0)	0 (0,0)	0.151
S amplitude PVC (mV)	0 (0,0)	0 (0,0)	0.519
QRS duration PVC (ms)	120 (112,120)	120 (112,123)	0.638
Lead aVL			
Q amplitude PVC (mV)	7.4 (5.9,9.2)	8 (0.63,11.38)	0.342
R amplitude PVC (mV)	0 (0,0)	0 (0,0.15)	0.005
S amplitude PVC (mV)	0 (0,0)	0 (0,0.6)	0.001
QRS duration PVC (ms)	108 (96,120)	114 (100,120)	0.114
Lead aVF			
Q amplitude PVC (mV)	0 (0,0)	0 (0,0)	0.121
R amplitude PVC (mV)	16.1 (13.5,18.5)	19.3 (15.73,22.7)	<0.001
S amplitude PVC (mV)	0 (0,0)	0 (0,0)	0.844
QRS duration PVC (ms)	116 (112,120)	120 (108,120)	0.561
Lead V1			
Q amplitude PVC (mV)	0 (0,0)	0 (0,0)	0.626
R amplitude PVC (mV)	1.2 (0.5,2.2)	1.8 (0.85,3.48)	0.021
S amplitude PVC (mV)	11.8 (8,15.2)	8.1 (5.05,11.58)	<0.001
QRS duration PVC (ms)	120 (104,124)	112 (100,123)	0.135
Lead V2			
Q amplitude PVC (mV)	0 (0,0)	0 (0,0)	0.131
R amplitude PVC (mV)	3.3 (2,5)	6.95 (4.73,10.08)	<0.001
S amplitude PVC (mV)	19 (13.8,24.2)	14 (10,19)	<0.001
QRS duration PVC (ms)	120 (120,128)	120 (112,128)	0.217
Lead V3			
Q amplitude PVC (mV)	0 (0,0)	0 (0,0)	0.361
R amplitude PVC (mV)	6.8 (4.8,9.1)	12.55 (9.53,19.53)	<0.001
S amplitude PVC (mV)	7.8 (2.4,13)	2.75 (0,8.88)	<0.001
QRS duration PVC (ms)	120 (120,128)	120 (112,128)	0.194
Lead V4			
Q amplitude PVC (mV)	0 (0,0)	0 (0,0)	1
R amplitude PVC (mV)	10.2 (8,13.7)	18.5 (12.98,25)	<0.001
S amplitude PVC (mV)	0 (0,0)	0 (0,0)	0.212
QRS duration PVC (ms)	120 (112,124)	120 (109,124)	0.214
Lead V5			
Q amplitude PVC (mV)	0 (0,0)	0 (0,0)	1
R amplitude PVC (mV)	14 (10.9,17)	20.4 (15.83,24.6)	<0.001
S amplitude PVC (mV)	0 (0,0)	0 (0,0)	0.186
QRS duration PVC (ms)	120 (112,124)	120 (109,120)	0.125
Lead V6			
Q amplitude PVC (mV)	0 (0,0)	0 (0,0)	1
R amplitude PVC (mV)	14.5 (12,17.1)	16.85 (14.85,21.75)	<0.001
S amplitude PVC (mV)	0 (0,0)	0 (0,0)	0.361
QRS duration PVC (ms)	120 (112,120)	118 (108,120)	0.146

**FIGURE 1:**
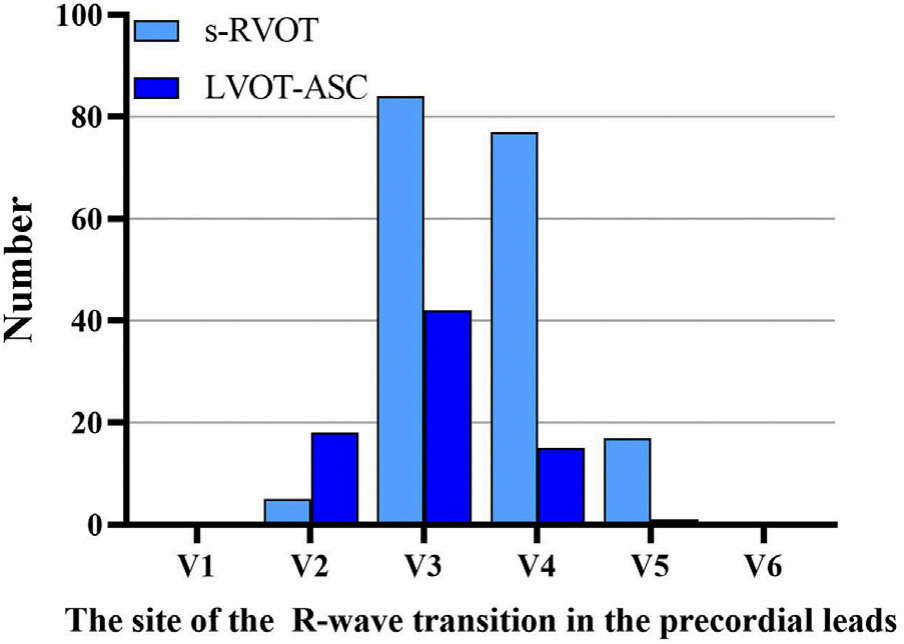
Sites of the R-wave transitions in the precordial leads in the s-RVOT and LVOT-ASC groups.

Of the 183 ECGs in the s-RVOT group, the most common pattern of V1 was the rS pattern (155 cases, 84.7%) followed by the QS pattern (28 cases, 15.3%). Of the 76 ECGs in the LVOT-ASC group, the pattern of the V1 lead was qrS in two cases (2.6%), QS in 12 cases (15.8%), and rS in 62 cases (81.6%). The most common transitional lead was V3 in both groups (Figure 1). Of the 183 PVCs in the s-RVOT group, 1.1%, 46.4%, 42.6%, and 9.8% had a transitional lead in leads V2, V3, V4, and V5, respectively. Meanwhile, 98.8% of the PVCs in the s-RVOT group had a transitional lead in V3, V4 and V5 lead. In the LVOT-ASC group, 76 PVCs were seen in the precordial transition leads V2–V5 (23.7%, 55.3%, 19.7%, and 1.3%, respectively), and 79.0% of the LVOT-ASC PVCs were observed in lead V3 and lead V2.

### A novel electrocardiographic criterion: The R-S difference index

**FIGURE 2:**
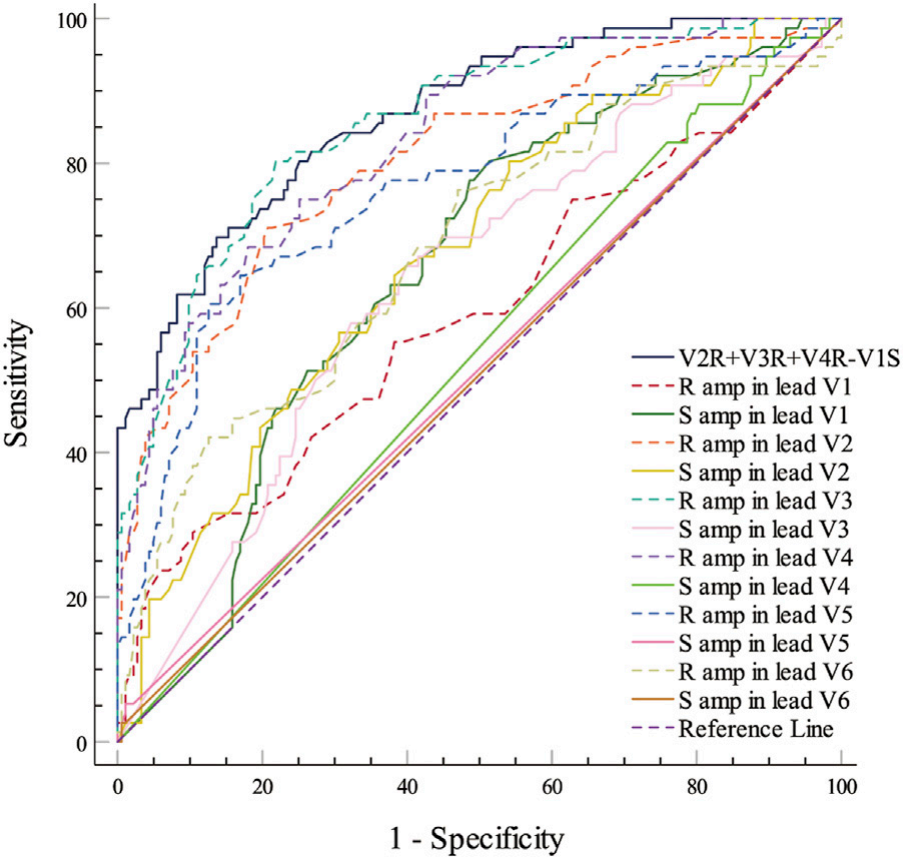
ROC curve analysis show the predictive accuracy of R-S difference index (V2R+V3R+V4R−V1S). R-S difference index was calculated from the R-wave amplitude in lead V3 with the greatest AUC of 0.0.856, followed by those in leads V4 and V2 (0.834 and 0.806, respectively). A joint logistic regression analysis model yielded an AUC value of 0.867 for V2R+V3R+V4R−V1S. ROC, Receiver operating characteristic; R amp, R-wave amplitude; S amp, S-wave amplitude.

**FIGURE 3:**
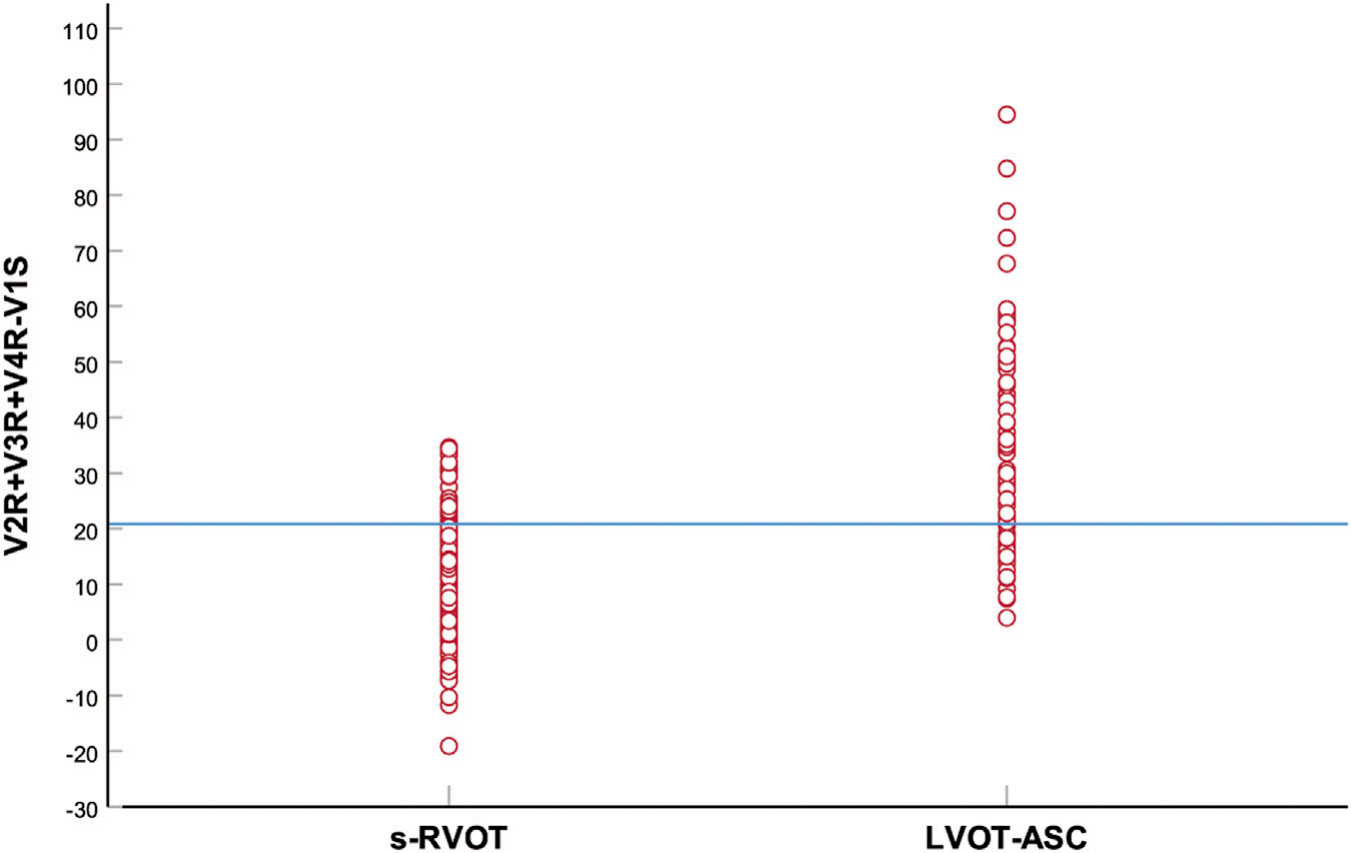
Scatter plot of the R-S difference index in the precordial leads of the s-RVOT and LVOT-ASC groups. The blue horizontal line indicates the optimal index cutoff value for differentiating s-RVOT and LVOT-ASC PVCs (20.9).

The PVCs of the two groups showed significant differences in R-wave amplitude in leads V1–V6 and S-wave amplitude in leads V1–V3. The R- and S-wave amplitudes in leads V1–V6 and the AUC values of the R-S difference index (calculated by V2R+V3R+V4R−V1S) in the precordial leads are shown in Figure 2. Among them, the AUC value of R-wave amplitude was largest in lead V3 (0.856) followed by leads V4 (0.834) and V2 (0.806). Based on a joint logistic regression model, the result of V2R + V3R + V4R − V1S had the largest AUC value of 0.867; thus, we defined this value as the R-S difference index. The optimal cutoff value of this index for differentiating s-RVOT and LVOT-ASC PVCs was 20.9; V2R+V3R+V4R−V1S > 20.9 indicates the PVCs originating from LVOT-ASC, on the contrary V2R+V3R+V4R−V1S ≤ 20.9 indicates PVCs from s-RVOT (Figure 3). The sensitivity and specificity of the R-S difference index were 73.7% and 86.3%, respectively. These results suggest that the R-S difference index is a reliable method to differentiate PVCs originating from s-RVOT and LVOT-ASC. Electrocardiographic measurements of the R-S difference index in precordial leads and the representative images of surface ECGs in both s-RVOT and LVOT-ASC groups were shown in Figure 5, 6.

### Comparison of the R-S difference index with current electrocardiographic methods for identifying premature ventricular contractions sites of origin

**FIGURE 4:**
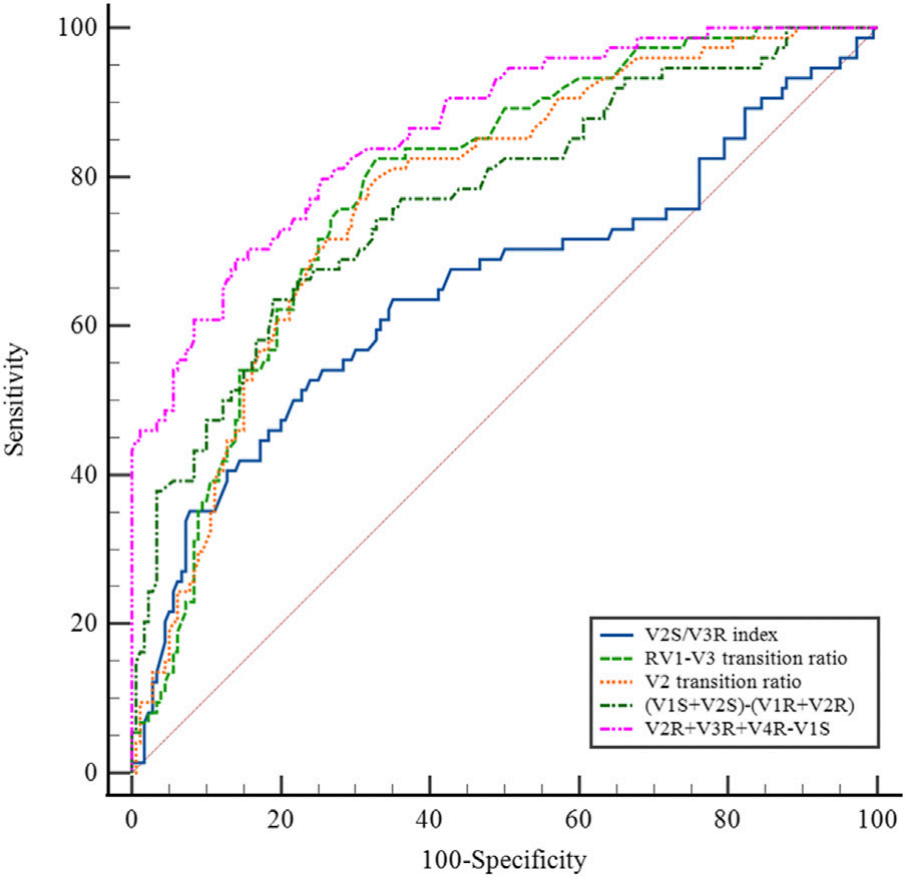
**(A)** Electrocardiographic measurements of the R-S difference index in precordial leads. **(A)** S-wave amplitude in lead 1 (mV); **(B)** R-wave amplitude in lead V2 (mV); **(C)** R-wave amplitude in lead V3 (mV); **(D)** R-wave amplitude in lead V4 (mV); The R-S difference index was calculated with the following formula: B+C+D-A.Fig.4B show the representative images of surface ECGs of both groups.

Several methods based on body-surface ECG have been proposed to identify PVCs originating from the left and right ventricular outflow tracts. These methods include the (i) V2S/V3R index (Yoshida et al., 2014); (ii) R-wave transition ratio in leads V1–V3 (RV1–V3 transition ratio (Efremidis et al., 2021), defined as the ratio of the sum of the R-waves in leads V1, V2, and V3 to the sum of the sinus R-waves in leads V1, V2, and V3); (iii) V2 lead transition ratio (Betensky et al., 2011); and (iv) difference in S-R wave amplitude in leads V1 and V2 [(V1S+V2S) − (V1R+V2R)] (Kaypakli et al., 2018). We calculated and compared the sensitivity and specificity of the above methods for identifying s-RVOT and LVOT-ASC PVCs and compared the sensitivity and specificity of the four methods mentioned above along with the R-S difference index proposed in this study. For the V2S/V3R index, the AUC value was 0.640, the optimal cutoff value was 1.44, and the sensitivity and specificity were 52.6% and 75.3%, respectively. For the R-wave transition ratio in leads V1–V3, the AUC was 0.786, the optimal cutoff value was 0.65, and the sensitivity and specificity were 82.4% and 67.2%, respectively. For the V2 lead transition ratio, the AUC was 0.775, the optimal cutoff value was 0.94, and the sensitivity and specificity were 78.9% and 67.2%, respectively. For the difference in S-R wave amplitude in leads V1 and V2, the AUC was 0.763, the optimal cutoff value was 15.7, and the sensitivity and specificity were 63.2% and 80.3%, respectively. In comparison with these four methods, the R-S difference index in the precordial leads had a higher AUC of 0.867 along with higher sensitivity and specificity (73.7% and 86.3%, respectively; Figure 4 and Table 3). Thus, the R-S difference index is a more reliable new method for identifying PVCs in the left and right ventricular outflow tract origin when the body-surface ECG shows PVCs with the LBBB pattern.

**FIGURE 5:**
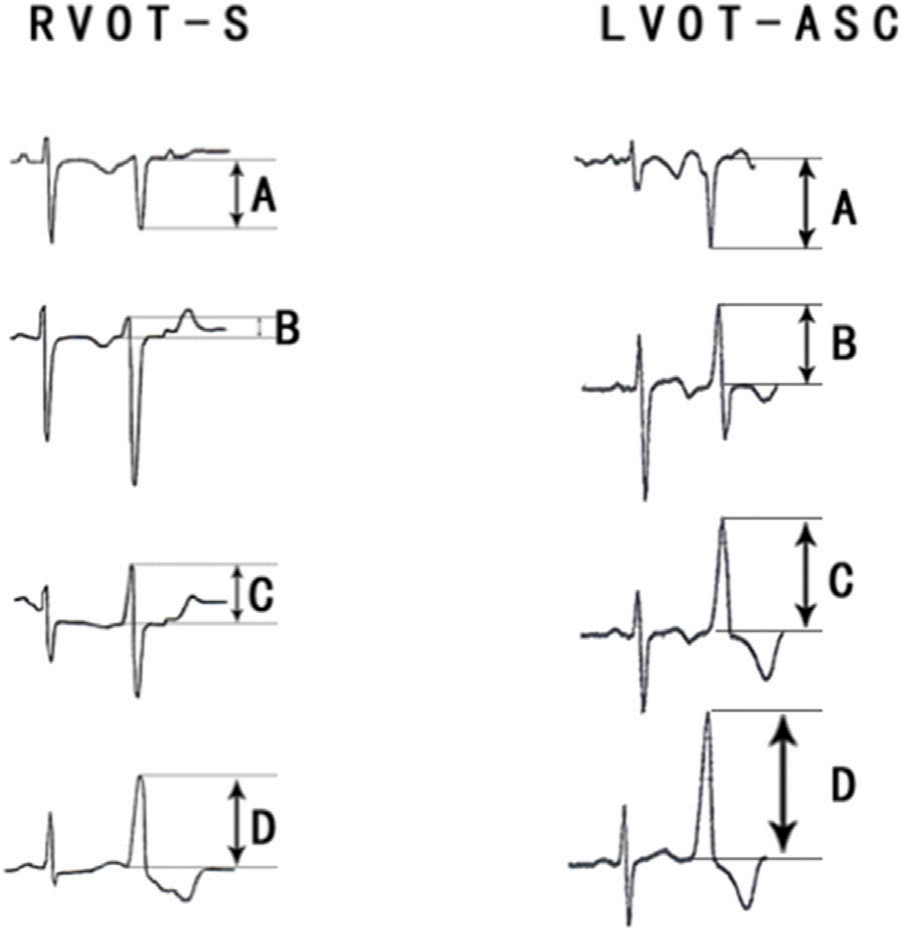
Comparison of the R-S difference index in the precordial leads with currently available ECG methods for identifying PVCs of the left and right ventricular outflow tracts.

**FIGURE 6:**
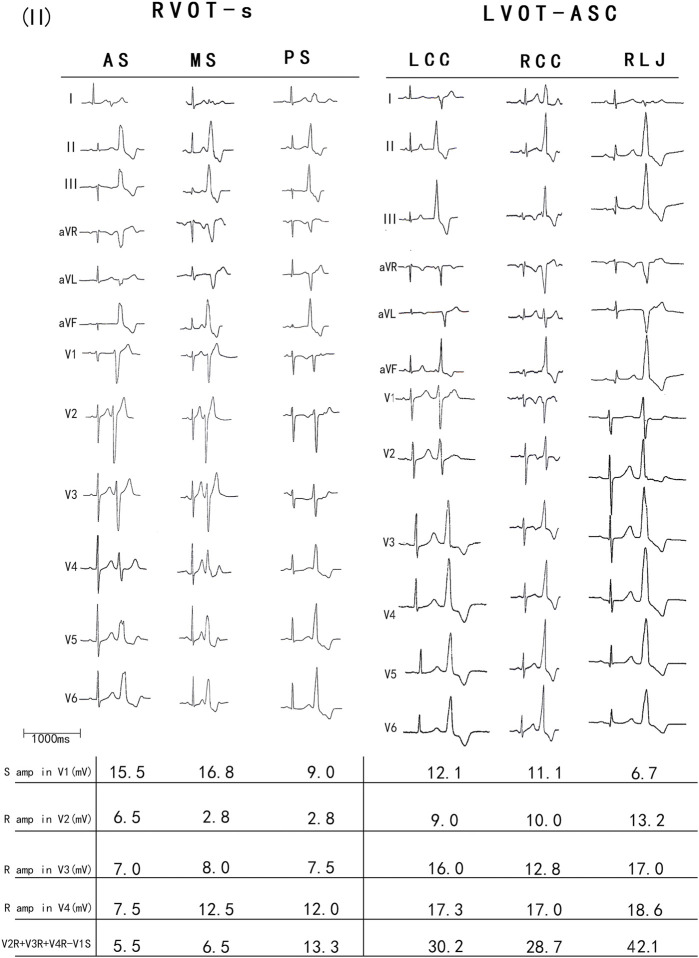
The representative images of surface ECGs and the R-S difference indexof both groups..

**TABLE 3:** Comparison of the novel ECG criterion with the previous ECG methods.

Indexes	Optimal cutoff value	AUC	Sensitivity	Specificity
V2S/V3R index	≤1.44	0.640	52.6	75.3
RV1–V3 transition ratio	>0.65	0.786	82.4	67.2
V2 transition ratio	>0.94	0.775	78.9	67.2
(V1S+V2S) − (V1R+V2R)	≤15.7	0.763	63.2	80.3
V2R+V3R+V4R−V1S	>20.9	0.867	73.7	86.3

## Discussion

PVCs originating from adjacent anatomical sites have similar body-surface ECG patterns. Understanding the anatomy of the s-RVOT and ASC is essential for the accurate preoperative determination of the site of origin of PVCs based on the ECG pattern. The anatomical locations of both sites causes RVOT PVCs to have smaller R-wave amplitudes of the precordial leads (especially leads V1–V3) and larger S-wave amplitudes of the V1 lead compared to LVOT-ASC PVCs. As a result, the RVOT PVCs show a smaller difference between the sum of the R-wave amplitudes of the precordial leads and the S-wave amplitude of the V1 lead. The findings of this study are consistent with the relationship between the anatomical characteristics of the s-RVOT and LVOT-ASC sites.

The septum of RVOT is adjacent to the LVOT-ASC. The posterior septum of the RVOT is adjacent to the RCC, and the anterior septum of the RVOT is next to the LCC. The above structures are adjacent, or even partially overlapping, in anatomical sites, resulting in similar body-surface ECG patterns of PVCs of the two sites mentioned above (Xiong and Zhu, 2020). The most common ECG manifestations of PVCs originating from the LVOT are an RBBB pattern in lead V1, a tall R-wave pattern in the inferior wall leads (II, III, and aVF), and a QS pattern in leads aVR and aVL. In contrast, the most common ECG presentation of the RVOT PVCs is a LBBB pattern in lead V1, a tall R-wave pattern in the inferior wall leads (II, III, and aVF), and a precordial transition lead, mostly in lead V3 and its posterior leads (Im et al., 2020). However, because of the anatomical proximity of the two sites, especially the proximity of the s-RVOT to the LVOT-ASC, some PVCs originating from the aortic sinus also exhibit the LBBB pattern. In this study, of the 133 cases of PVCs in the aortic sinus (69 in the LCC, 39 in the RCC, and 25 in the left-right sinus junction) in which radiofrequency ablation was successfully performed, 76 (57.1%) had an LBBB pattern, and the thoracic migrating leads were all leads V2–V5, will lead V3 being the most common (55.3%) followed by lead V2 (23.7%). Of the 275 RVOT PVCs (87 at the free wall site and 188 at the septal site), 272 (98.9%) had a LBBB pattern in lead V1, and the most common precordial transition lead was V3 (42.9%) followed by lead V4 (42.5%). Thus, the ECGs of both s-RVOT and LVOT-ASC PVCs can show an LBBB pattern in lead V1 and a shift in lead V3. Therefore, in clinical work, the identification of PVCs originating from the left and right ventricular outflow tracts based on the patterns of the V1 leads, the direction of the QRS axis, and the characteristics of the transition leads to a high rate of misclassification. Di et al. (2019) analyzed the electrocardiographic indices of 147 PVCs of the left and right ventricular outflow tracts, both with the transition lead in V3. They concluded that the V1–V3 transition index can be used to differentiate PVCs of the RVOT and LVOT with the transition lead in V3 (AUC = 0.932, optimal cutoff value = −1.60). The sensitivity and specificity of the V1–V3 transition index for predicting PVCs in the RVOT were 93% and 86%, respectively. Although the discriminatory value of the V1–V3 transition index is high, the calculation is complex, limiting its clinical applications. Efremidis et al. (2021) studied the same subjects with PVCs at the outflow tract site in the shifted V3 leads and obtained an AUC of 0.856 for the R-wave shift ratio (RV1–V3 transition ratio) in leads V1–V3, an optimal cutoff value of 0.9 to predict PVCs in the LVOT, and a sensitivity and specificity of 94% and 73%, respectively. The identification of PVCs in the left and right outflow tract sites in V1 lead exhibiting LBBB patterns has also been studied by several authors. Yoshida et al. (2011) analyzed the ECG patterns of 112 PVC cases in the outflow tract with LBBB patterns (87 in the RVOT and 25 in the LVOT-ASC). They found that the transition zone index (cutoff value = 0) had a sensitivity and specificity of 88% and 82%, respectively, for LVOT-ASC PVCs. This index excludes the influence of individual differences in cardiac rotation on PVC patterns. While it has a high predictive value, the relatively complex calculation method limits its widespread use in clinical work. In this study, we compared V2S/V3R index, the R-wave transition ratio in leads V1–V3 (RV1–V3 transition ratio), the V2 lead shift ratio, the difference in S-R wave amplitude in leads V1–V2, and the R-S difference index in the precordial leads for differentiating s-RVOT and LVOT-ASC PVCs with the LBBB pattern. Among the methods, the R-S difference index had the highest AUC value. Thus, the R-S difference index in the precordial leads is a reliable method for identifying PVCs in specific sites of the outflow tract (specifically the s-RVOT and ASC) when the ECG shows an LBBB pattern with inferior axis.

Radiofrequency catheter ablation is a common treatment strategy for idiopathic PVCs because of its high success rate and good results (Oomen et al., 2018). PVCs in outflow tract are the most common types of idiopathic PVCs. The ECG morphology patterns of PVCs with different origin have specific characteristics. However, PVCs that are anatomically adjacent show similar ECG characteristics. For example, the ECG patterns of PVCs at the RVOT posterior septum and LVOT-RCC sites have the following characteristics: (i) LBBB pattern in lead V1; (ii) tall R-wave pattern in the inferior wall leads (leads II, III, and aVF); and (iii) the most common precordial shifted lead is V3. Therefore, it is difficult to distinguish PVCs originating from the LVOT and RVOT based only on characteristics such as the bundle branch pattern in V1 and the precordial shift leads. The preoperative determination of the PVC origin by ECG can help the operator quickly and accurately identify the target of ablation, thus optimizing the surgical procedure, shortening the operative time, reducing puncture injuries, and reducing the incidence of postoperative complications such as hematoma and arteriovenous fistula. In this study, we demonstrated that the R-S difference index, which is simple to calculate, can help electrophysiologists quickly and accurately locate the target site preoperatively. Thus, this method has promising clinical applications.

Limitations: First, this was a single-center retrospective study with a limited sample size. Future trials with larger sample sizes are needed to validate the predictive value of this method. Second, the samples included in this study were only a subset of patients with PVCs in the outflow tract who underwent successful radiofrequency ablation, and only ECG indices of PVCs in the s-RVOT and LVOT-ASC (LCC, RCC, and left-right coronary sinus junction) sites were analyzed. Thus, the predictive value of the R-S difference index for other sites of the left and right ventricular outflow tracts, including the RVOT-free wall, pulmonary artery, AMC area, and epicardial sites, needs to be further confirmed.

## Summary

The QRS patterns and amplitudes in each lead of the ECGs of 259 PVC cases in the outflow tract with the LBBB pattern and inferior axis were statistically analyzed. The R-S difference index in the precordial leads was found to be a reliable method for predicting PVCs in the s-RVOT and LVOT-ASC before ablation. The preoperative determination of the PVC site is clinically important to optimize the surgical procedure, shorten the operative time, and reduce the incidence of puncture-related complications.

## Data Availability

The raw data supporting the conclusions of this article will be made available by the authors, without undue reservation.
